# Influence of Straw Burning on Urban Air Pollutant Concentrations in Northeast China

**DOI:** 10.3390/ijerph16081379

**Published:** 2019-04-17

**Authors:** Zhenzhen Wang, Jianjun Zhao, Jiawen Xu, Mingrui Jia, Han Li, Shijun Wang

**Affiliations:** 1 School of Geographical Sciences, Northeast Normal University, Changchun 130024, China; wangzz280@nenu.edu.cn (Z.W.); xujw685@nenu.edu.cn (J.X.); jiamr884@nenu.edu.cn (M.J.); lih317@nenu.edu.cn (H.L.); 2 Key Laboratory of Geographical Processes and Ecological Security in Changbai Mountains, Ministry of Education, Northeast Normal University, Changchun 130024, China; 3 Urban Remote Sensing Application Innovation Center, Northeast Normal University, Changchun 130024, China

**Keywords:** pollutant concentration, straw burning, fire spots, PM_2.5_, PM_10_, AQI

## Abstract

Northeast China is China’s primary grain production base. A large amount of crop straw is incinerated every spring and autumn, which greatly impacts air quality. To study the degree of influence of straw burning on urban pollutant concentrations, this study used The Moderate-Resolution Imaging Spectroradiometer/Terra Thermal Anomalies & Fire Daily L3 Global 1 km V006 (MOD14A1) and The Moderate-Resolution Imaging Spectroradiometer/Aqua Thermal Anomalies and Fire Daily L3 Global 1 km V006 (MYD14A1) data from 2015 to 2017 to extract fire spot data on arable land burning and to study the spatial distribution characteristics of straw burning on urban pollutant concentrations, temporal variation characteristics and impact thresholds. The results show that straw burning in Northeast China is concentrated in spring and autumn; the seasonal spatial distributions of PM_2.5_, PM_10_ andAir Quality Index (AQI) in 41 cities or regions in Northeast China correspond to the seasonal variation of fire spots; and pollutants appear in the peak periods of fire spots. In areas where the concentration coefficient of rice or corn is greater than 1, the number of fire spots has a strong correlation with the urban pollution index. The correlation coefficient *R* between the number of burned fire spots and the pollutant concentration has a certain relationship with the urban distribution. Cities are aggregated in geospatial space with different *R* values.

## 1. Introduction

As a large agricultural country, China’s straw crop output reached 700 million tons in 2013 [1]. Northeast China is China’s main grain production area. In 2015, the straw production in Northeast China was approximately 159 million tons, accounting for 19.2% of the country’s total straw production [2]. Pollutants produced by biomass burning have become a major source of air pollution [3,4,5], and straw open burning is an important contributor. Straw burning has seasonal and cyclical changes [6]. Increasing evidence shows that straw burning has become an important factor affecting urban air quality [7]. Straw burning strongly affects the atmospheric environment quality, resulting in a significant increase in the total amount of suspended particulate matter in the air, and this burning produces a large amount of harmful gases, such as PM_2.5_ and PM_10_ [7,8]. The pollutants produced by straw open burning have a long atmospheric residence time and are one of the major pollution sources in this region and the world [9]. In addition, open-air straw burning causes a series of traffic and safety problems [6]. In recent years, the pollutant weather in the spring and autumn in the northeastern area has been frequent, with a wide range of influences, which has caused widespread concern [10].

In recent years, scholars have conducted studies on the weather caused by straw burning and have obtained a series of research results. Some scholars found that open-air biomass burning significantly impacts urban air quality by monitoring biomass produced by forests and farmland biomass burning in southeastern Queensland, Australia [7]. Under high relative humidity and south winds, the pollutant emissions from wheat straw burning in northern China and the high pollutant emissions from cities and industries have led to increased pollution in the North Plain [11]. Open burning of crop straw has important environmental impacts including reducing visibility, worsening air quality (sometimes severe smog pollution) and endangering public health [12]. A severe atmospheric haze event in Changchun city was caused by strong source emissions (such as biomass burning) and unfavourable air diffusion conditions. Among the five emission factors in Changchun, biomass burning accounted for 20% [13]. Satellite technology has also been used in national straw burning monitoring [14]. The results show that the change trend of fire spots in the buffer zone of some cities in the 700–800 km range is in good agreement with the air pollution index, and the correlation coefficient is 0.54 [14].

Traditional straw burning monitoring methods (such as point-by-point manual inspection) have the disadvantages of low efficiency, low coverage, and high costs. Satellite remote sensing has enabled quick and extensive burning monitoring due to its timeliness, wide coverage and high resolution [15]. The Moderate-Resolution Imaging Spectroradiometer (MODIS) is an advanced multi-spectral remote sensing sensor with 36 observation channels covering the main observations of the current major remote sensing satellites. Among these channels, MODIS/Terra Thermal Anomalies & Fire Daily L3 Global 1 km V006 (MOD14A1) and MODIS/Aqua Thermal Anomalies and Fire Daily L3 Global 1 km V006 (MYD14A1) thermal anomaly product data can be used directly, which is an ideal data source for monitoring straw burning.

Based on MOD14A1 and MYD14A1 thermal anomaly product data, surface cover change data and pollutant monitoring site data in Northeast China, this study explores the distribution characteristics and relationship of straw burning with urban pollutant concentrations in time and space, conducts in-depth research on rice and corn concentrated planting areas, and performs a quantitative analysis of typical cities. The research results can be used to implement policies prohibiting straw burning by government departments at all levels, to establish a limited burning zone for straw burning zones, to generate scientific support for controlling straw burning, and to aid in monitoring and early warning of pollutant disasters. The factors that affect the serious pollution in the spring and autumn seasons in Northeast China have been determined to aid the development of straw harmless treatment technology at the source, to contribute to maintaining people’s health and to improve the ecological environment.

The research objectives of this paper are as follows: (1) Explore the temporal and spatial distribution of straw burning fire spots and 41 city or regional pollutant concentrations in Northeast China. The relationship between straw burning and pollutant concentrations has been explored on temporal and spatial scales; (2) study the degree of influence of straw burning on pollutants in urban centres at different radius buffers and explore the maximum threshold value of straw burning on urban pollutant concentrations; (3) explore the relationship between rice- and corn-concentrated planting areas and fire spots and pollutants, and quantitatively study the extent of the impact of fire spots around typical cities in Northeast China on the city pollutants.

## 2. Materials and Methods

### 2.1. Study Area

**Figure 1:**
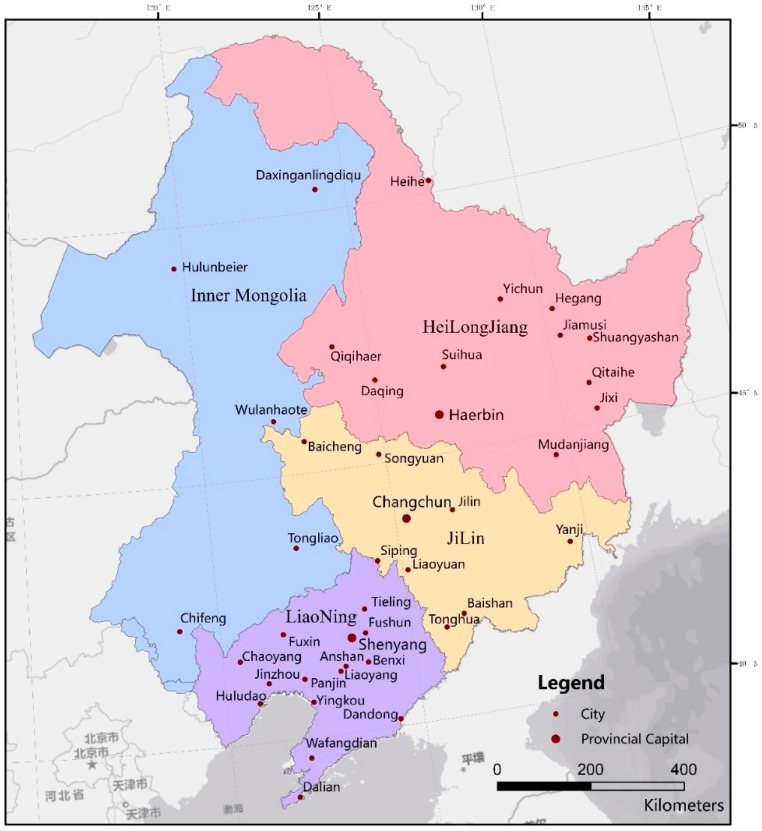
Map of the study area.

The northeastern region includes Heilongjiang Province, Jilin Province, Liaoning Province and Hulunbeier, Wulanhaote, Tongliao and Chifeng cities in the eastern part of the Inner Mongolia Autonomous Region, located at 115°05′–135°02′E, 38°40′–53°34′N [16] (Figure 1). The northeastern region has a continental monsoon climate, with mild and humid summers and long cold winters [16]. The annual average temperature is 2.75–5.72 °C, the average annual precipitation is 250–700 mm, the accumulated temperature above 10 °C is 1500–3700 °C, and the frost-free period is 90–160 days. Corn, soybean, rice and spring wheat are grown [16]. The yield of crop straw and the density of straw resources are high [17]. The main straw-burning products in Northeast China are corn and rice.

### 2.2. Data

This study used MOD14A1 and MYD14A1 data for 2015–2017 with a spatial resolution of 1 km to extract straw-burned fire spots. The land cover type data were derived from the surface coverage change data of MCD12Q1 (Land Cover Type Yearly L3 Global 500 m SIN Grid) with a spatial resolution of 500 m, which was used to extract agricultural land in the study area. MOD14A1 and MYD14A1 products are based on the 3.9 μm and 11 μm bands of MODIS sensors on the Terra satellite and Aqua satellite, respectively. By setting the thresholds for fire spots and background temperature difference, the MODIS research team extracts the temperature anomaly area and fire spot data for day and night. The following table is the metadata description of the FireMask data set of MOD14A1 and MYD14A1 products. Table 1 shows that the pixel values 7, 8 and 9 represent the fire spot data with low confidence, nominal confidence and high confidence, respectively, and these values are all identified as fire spot data in this study.

The pollutant data were derived from the China Air Quality Online Monitoring and Analysis Platform [18]. This research mainly used Air Quality Index (AQI), PM_2.5_ and PM_10_ data. Pollutant data for 41 cities in the study area were selected from March 2015 to March 2017.

### 2.3. Methods

#### 2.3.1. Extraction and Treatment of Burning Straw Data

In the MODIS thermal anomaly and fire product data, the values of “7, 8, 9” are fire spots; they were reassigned as 1, and the other values are non-fire spots, which were assigned a value of 0. The MOD14A1 and MYD14A1 data from the same period were combined, and the obtained data only contains the fire spot and non-fire spot data. The crop area in the MCD12Q1 land cover type data at 500-m resolution was used as a mask to extract the fire spots in the agricultural land to determine the number of fire spots of crop straw burning in the northeastern region for each day.

The spring, summer, autumn and winter seasons in this study were divided according to “March, April, May”, “June, July, August”, “September, October, November”, and “December, January (the next year), February (the next year)”. According to the seasonal superposition of the fire spots in 2015 and 2016, fire spot spatial distribution maps of the four seasons of spring, summer, autumn and winter were obtained, and the annual data were superimposed to obtain the annual fire spot distribution map.

#### 2.3.2. Contaminant Data Acquisition and Processing

The daily monitoring data of pollutants, such as PM_2.5_, PM_10_ and AQI, in 41 cities or regions in Northeast China were processed to obtain the AQI, PM_2.5_ and PM_10_ in the four seasons and the whole year for all cities in 2015 and 2016. The average value was interpolated in the Kriging space to obtain a spatial distribution map of the seasonal and annual air pollution concentrations in Northeast China.

#### 2.3.3. Correlation Analysis

We counted the number of fire spots in different buffer zones in different cities, summed the fire spots and urban pollutant data in the buffer every 8 days, and then conducted correlation analysis to obtain the correlation coefficient between the fire spots in different buffers and the pollutants in the central city. A two-dimensional map of the correlation coefficients between the fire spots in different buffers and the pollutants in the central city was obtained.

#### 2.3.4. Selecting Typical Cities for Quantitative Analysis

To analyse the quantitative relationship between fire spots and pollutants, some typical cities were selected for analysis. The selection of typical cities considered three conditions: (1) From March 2015 to March 2017, the number of straw burning points in a 150-km radius was ranked as the top 16 (number of fire points greater than 1700); (2) PM_2.5_, PM_10_, AQI and the correlation coefficient of the number of burned fire spots in different buffer zones were evaluated, and cities with *R* values greater than 0.5 for two or more pollutants were selected; (3) considering that the provincial capital has a large population, industrial development and many vehicles, the provincial capitals were also considered typical cities. Finally, eight typical cities including Harbin, Changchun, Shenyang, Songyuan, Shuangyashan, Jiamusi, Qitaihe and Suihua were selected.

## 3. Results

### 3.1. Analysis of Straw Fire Spots

#### 3.1.1. Analysis of the Temporal Distribution of Straw Fire Spots

**Figure 2:**
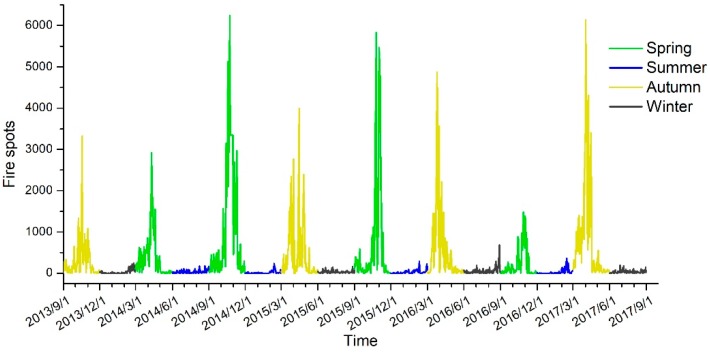
Straw fire spots in Northeast China from 2013 to 2016.

**Figure 3:**
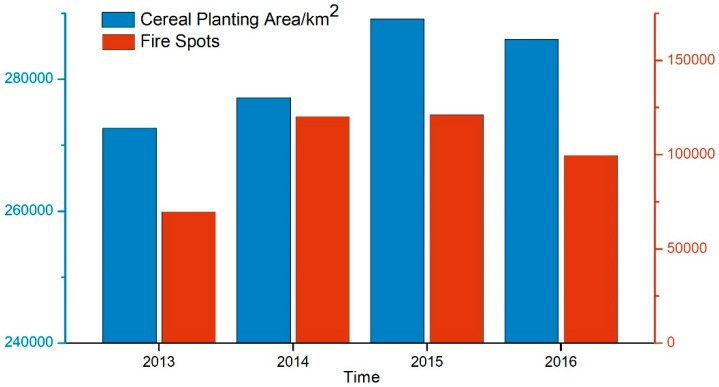
Change in the total number of fire spots and cereal planting area in Northeast China from 2013 to 2016.

According to the situation of farmers burning straw in the northeastern region, the straw obtained in the autumn harvest was burned in the autumn of that year and the spring of the following year. Here, a straw burning year is defined from September 1 of the current year to August 31 of the following year. According to our statistics, the total number of fire points in the burning years of 2013, 2014, 2015 and 2016 was 71,599, 122,137, 123,131 and 101,588, respectively. From 2013 to 2016, the graph of the number of fire spots in Northeast China (Figure 2) shows that within a straw burning year, the number of fire spots was highest in spring and autumn. The number of fire spots generally increased first and then decreased, which was consistent with the change of the cereal planting area in Northeast China. From 2013 to 2016, the cereal planting area in Northeast China peaked in 2015 and then gradually decreased (Figure 3).

#### 3.1.2. Spatial Distribution of Straw Fire Spots

**Figure 4:**
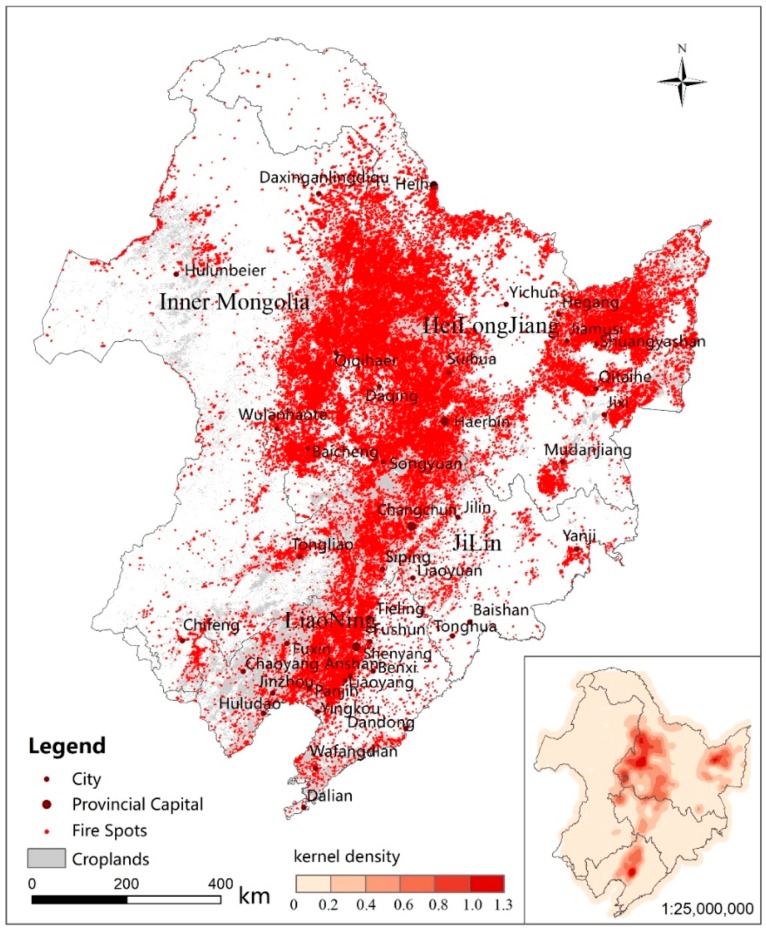
Spatial distribution map of fire points from 2015 to 2016.

The fire spots in 2015 and 2016 were summed to obtain a fire spot distribution map for 2 years. We recorded the frequency of fire occurrences in 2 years and used nuclear density analysis to obtain a frequency map of the fire spots. The combination of the two figures resulted in Figure 4. In Figure 4, the fire spots of straw burning are distributed along a large area of cultivated land. The straw fire spots are concentrated in the southwestern (Harbin, Daqing, Qiqihar, and Suihua) and northeastern (Shuangyashan, Jiamusi, and Hegang) parts of Heilongjiang Province, northwestern (Baicheng) and central (Changchun and Songyuan) Jilin Province, and central Liaoning Province (Shenyang, Anshan, Liaoyang, and Panjin). Among these areas, the frequency of fire spots in the southwestern part of Heilongjiang Province and the northeastern part of Liaoning Province is higher than that of other areas, followed by the central and western parts of Jilin Province.

**Figure 5:**
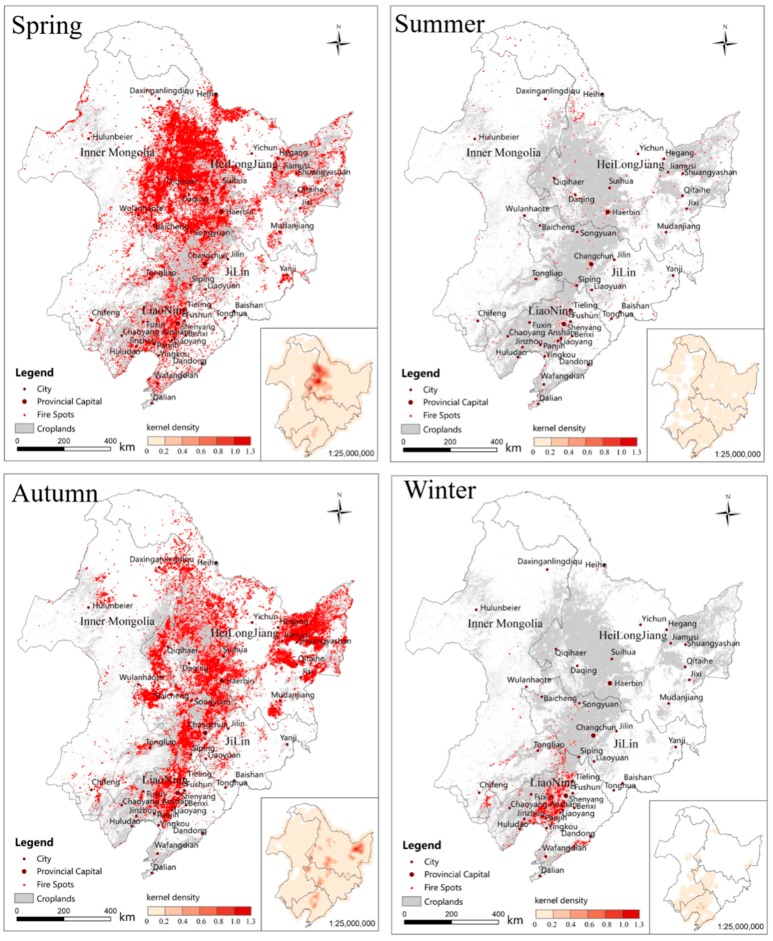
Distribution of fire spots in different seasons for 2015 and 2016.

The fire points in the spring, summer, autumn and winter seasons of 2015 and 2016 were summed to obtain the fire spot distribution maps in different seasons (Figure 5). We recorded the frequency of fire occurrences in different seasons for 2 years and used nuclear density analysis to obtain the frequency of occurrence of fire spots (Figure 5). In Figure 5, the number of fire points is the highest in spring and autumn and the lowest in summer and winter. The fire spots in spring are mainly distributed in Harbin, Daqing, Qiqihar and Suihua in southwestern Heilongjiang Province. The fire spots in autumn are mainly distributed in Shuangyashan, Jiamusi and Hegang in northeastern Heilongjiang Province; Baicheng in northwestern; Changchun and Songyuan in central Jilin Province; and Shenyang, Anshan, Liaoyang and Panjin in central Liaoning Province. The fire spots in winter are mainly distributed in Liaoning Province, which is related to the low latitude of Liaoning Province. This low latitude causes the snow cover time in Liaoning Province to be shorter in winter, which leads to straw burning in winter.

### 3.2. Analysis of Pollutants

#### 3.2.1. Analysis of Pollutants on a Temporal Scale

**Figure 6:**
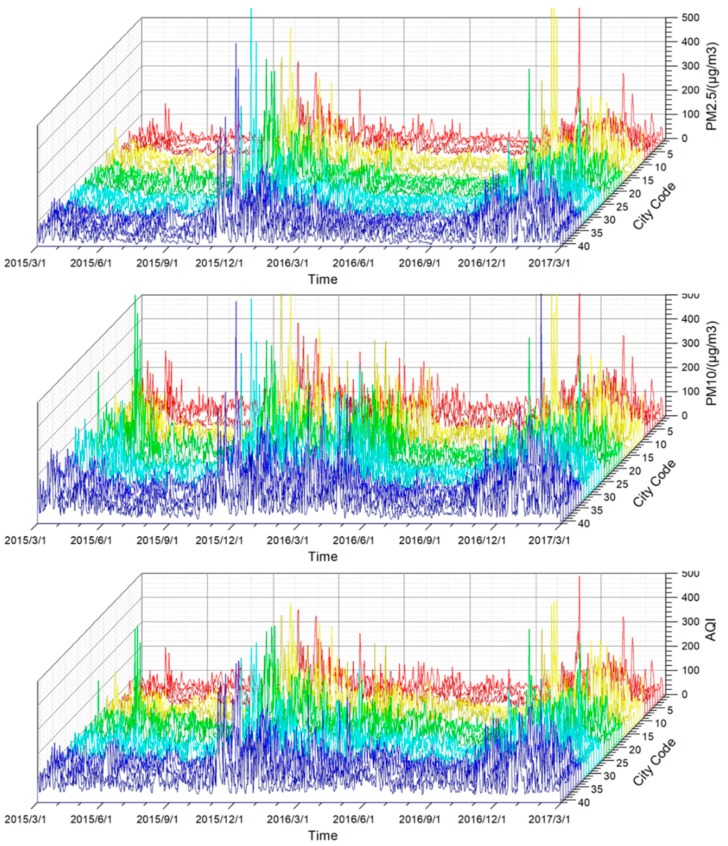
Pollutant concentration changes in 41 cities or regions in Northeast China (x-axis is the time axis; y-axis is the city code; and z-axis is the PM_2.5_, PM_10_, and AQI levels).

Three parameters of pollutants, AQI, PM_2.5_ and PM_10_, from March 2015 to March 2017 were selected. Figure 6 shows that, in the 41 cities or regions in Northeast China, PM_2.5_, PM_10_ and AQI peaked in autumn and spring in 2015 and 2016. The 2015 and 2016 air quality parameters (AQI, PM_2.5_ and PM_10_) show that the air pollution in most cities in 2015 was the most serious in early November, followed by December; the air pollution in most cities in 2016 was the most serious in December, followed by the first half of January in the following year. There are obvious time series differences between 2015 and 2016. Figure 2 shows that the number of fire spots was highest in autumn 2015. The obvious pollutant concentration caused by straw burning in autumn may cause a serious air quality decline in November.

The 41 cities or regions in Northeast China are coded in the order of north to south and west to east (Table 2).

#### 3.2.2. Analysis of Pollutants on a Spatial Scale

**Figure 7:**
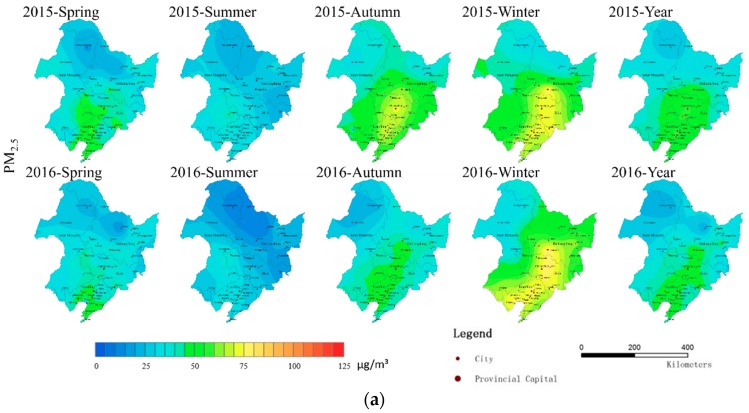

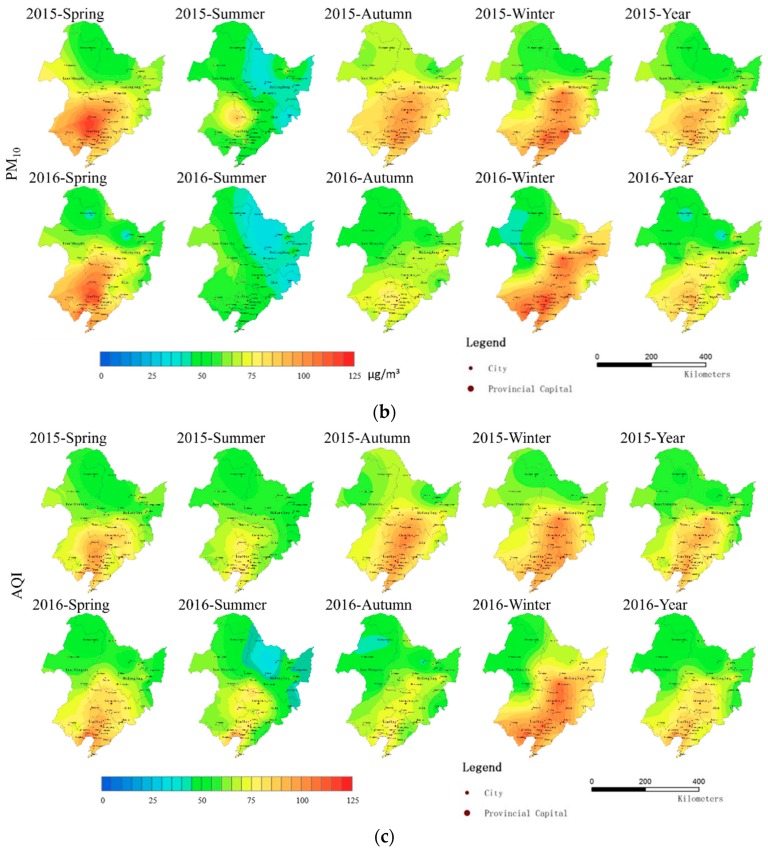
(**a**) PM_2.5_, (**b**) PM_10_ and (**c**) AQI spatial interpolation maps for different seasons in 2015 and 2016.

The spatial distribution map of PM_2.5_, PM_10_ and AQI (Figure 7) shows that the air pollution concentration in the four seasons in Northeast China is quite different; winter and spring are more serious, summer is good, and autumn shows differences. The pollutant concentration in autumn 2015 was significantly higher than that in 2016. The spatial distribution map of the annual average pollutants shows high pollutant concentrations in Harbin, Changchun and Shenyang. The concentration of air pollutants in urban areas with the three capital cities of Shenyang–Changchun–Harbin as the axis is significantly higher than that in other cities. Harbin, Changchun and Shenyang show a clear diffusion trend.

The fire spot distribution map (Figure 2) and the spatial distribution maps of PM_2.5_, PM_10_ and AQI in different seasons and in 2015–2016 (Figure 7) show that the pollutant concentration corresponding to spring and autumn concentrations in the fire spots is significantly higher than that in summer but lower than the pollutant concentration in winter. The PM_2.5_, PM_10_ and AQI pollutant concentrations in the autumn of 2016 were all lower than that in the autumn of 2015 and lower than that in the spring of 2016. The number of fire spots in 2016 was significantly lower than that in 2015, indicating that the number of fire spots in the autumn of 2015 led to an increased pollutant concentration in the autumn of 2015, and the decreased number of straw-burning fire spots in the autumn of 2016 made the concentrations of PM_2.5_, PM_10_ and AQI in the autumn of that year lower than the pollutant concentrations in the same period in 2015.

### 3.3. Relationship between the Number of Fire Spots and The Pollutant Concentration in Different City Buffer Zones

In the range of 10–300 km in 41 cities or regions in Northeast China, the cumulative radius of 10 km was used to study the correlation between the number of fire spots in different radius buffers and the PM_2.5_, PM_10_ and AQI in the central city to determine the impact of straw burning on urban pollutants. We found that the impact of straw burning on urban pollutants has much to do with the geographical distribution of cities.

**Figure 8:**
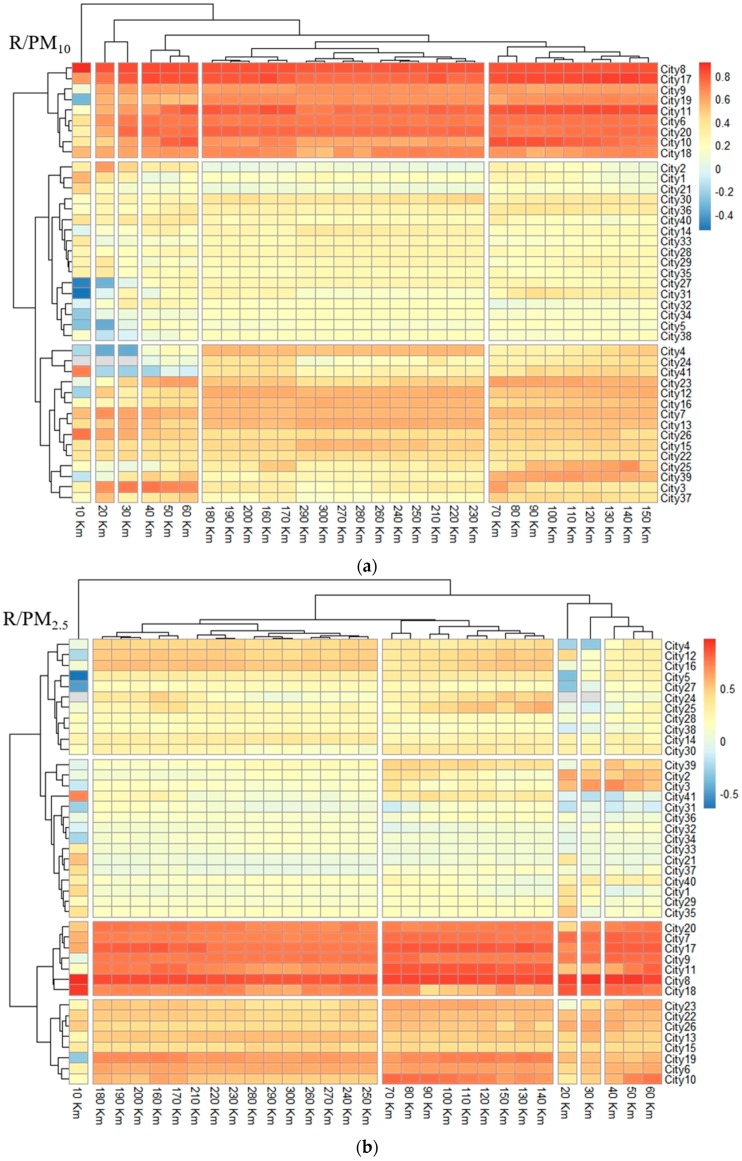

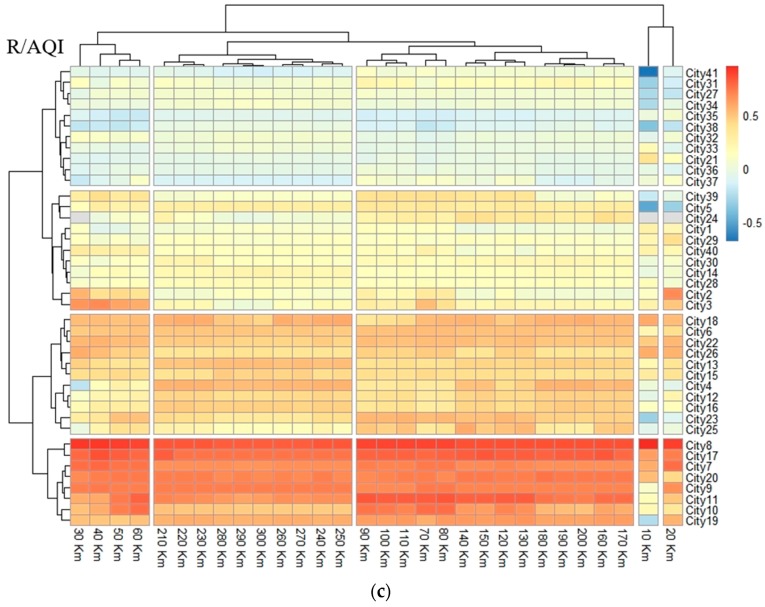
Two-dimensional map of the correlation between the number of fire spots and the (**a**) PM_2.5_, (**b**) PM_10_ and (**c**) AQI in the 10–300 km radius buffer zone of 41 cities or regions in Northeast China (the abscissa is the radius; the ordinate is the city number).

**Figure 9:**
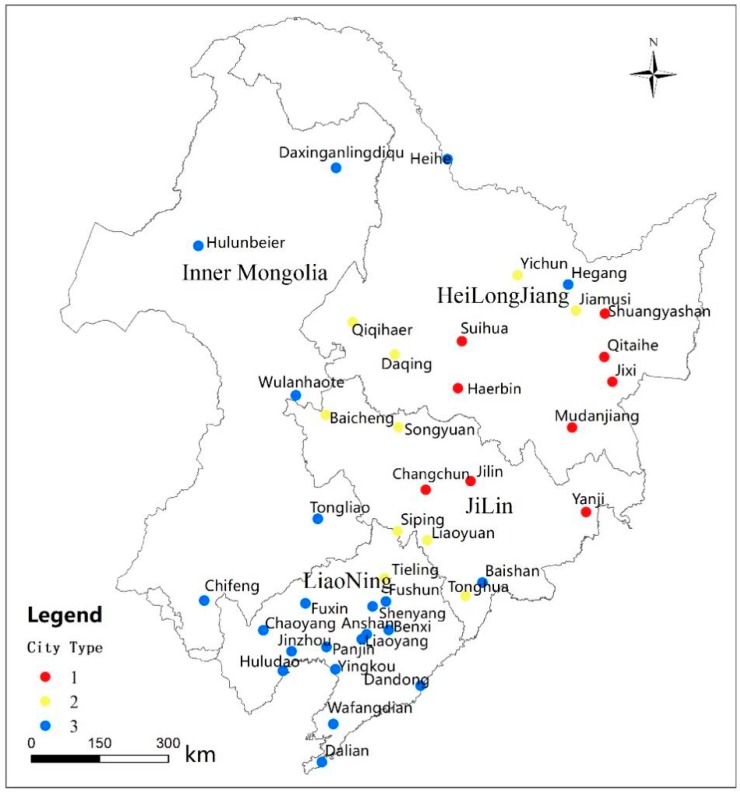
Distribution of different types of cities.

Within the results of the radius cluster analysis (Figure 8), the results of the cluster analysis of the radii of PM_2.5_ and PM_10_ are completely consistent. Among them, the radii of 10 km, 20 km and 30 km are each of the same type, the radius from 40 km to 60 km is one type, the radius of 70 km–150 km is another type, and the radius of 160 km–300 km is another type. AQI clusters of radii are divided into five categories. Among them, the radii of 10 km and 20 km are one class each, the radius from 30 km to 60 km is one class, the radius of 70 km–200 km is one class, and the radius of 210 km–300 km is one class. The effect of straw burning on pollutants has a certain relationship with the radius. From the results of the city cluster analysis (Figure 8), PM_2.5_ and AQI have been divided into four categories, and PM_10_ has been divided into three categories. The first category of *R* values in the three pollutant clustering results is large, and the second category has lower *R* values. The third and fourth categories of PM_2.5_ and AQI and the third category of PM_10_ have the lowest *R* values. We selected all cities that meet at least two of the three largest *R* values of PM_2.5_, PM_10_ and AQI. These cities are numbered from 7 to 11 and from 17 to 20. We defined them as city type 1. For example, city number 7 is located in the category in which PM_2.5_ and AQI have the largest *R* value in the city clustering result. Similarly, we selected all cities that meet at least two of the lower *R* values of PM_2.5_, PM_10_, and AQI. These city numbers are 4, 6, 12, 13, 15, 16, 22, 23, 25 and 26. We defined these numbers as city type 2. The remaining cities are located in the third and fourth categories of PM_2.5_ and AQI and in the third category of PM_10_. We defined these cities as city type 3. Different types of cities are geographically clustered (Figure 9).

### 3.4. Analysis of the Relationship between Fire Spots and Pollutants in Concentrated Planting Areas of Corn and Rice

The concentration coefficient indicates the ratio of the per capita output value (or output) of a certain regional industry to the corresponding per capita output value (or output) of the country. If the concentration coefficient is greater than 1, that crop type is concentrated in the region and the degree of specialization is high [19]. Previous studies have indicated that the cities with a rice concentration coefficient greater than 1 in the three northeastern provinces are: Jiamusi, Shuangyashan, Jixi, Hegang, Yichun, Harbin, and Baicheng; cities with a corn concentration coefficient greater than 1 include Jiamusi, Shuangyashan, Hegang, Harbin, Suihua, Qiqihar, Daqing, Baicheng, Songyuan, Changchun, Siping, Liaoyuan, and Fuxin [19]. Using the 150-km radius as the threshold, a city is the centre within the radius of 150 km. The cities with more than 1700 fire spots from March 2015 to March 2016 were Daqing, Qiqihar, Harbin, Suihua, Songyuan, Shuangyashan, Shenyang, Liaoyang, Anshan, Fushun, Tieling, Benxi, Jiamusi, and Qitaihe for a total of 16. Among these cities, eight have corn or rice concentration coefficient greater than 1. As a result of the correlation coefficient between the cities’ PM_2.5_, PM_10_, AQI and the number of fire points in different radii, there are 14 cities that meet the condition that the correlation coefficient between at least two types of pollutants and the number of fire spots is greater than 0.5. These cities are Qitaihe, Jixi, Mudanjiang, Shuangyashan, Baicheng Suihua, Jilin, Changchun, Songyuan Yanji, Harbin, Jiamusi, Liaoyuan, Siping, Hegang Songyuan and Yichun. Among them, 10 have a rice or corn concentration coefficient greater than 1. This finding shows that, with more concentrated rice and corn planting around a city in the northeastern region, the more fire spots are generated by straw burning and the greater the impact on the central city.

### 3.5. Typical City Analysis

#### 3.5.1. Changes in the Number of Fire Spots in Different Buffers of Typical Cities

**Figure 10:**
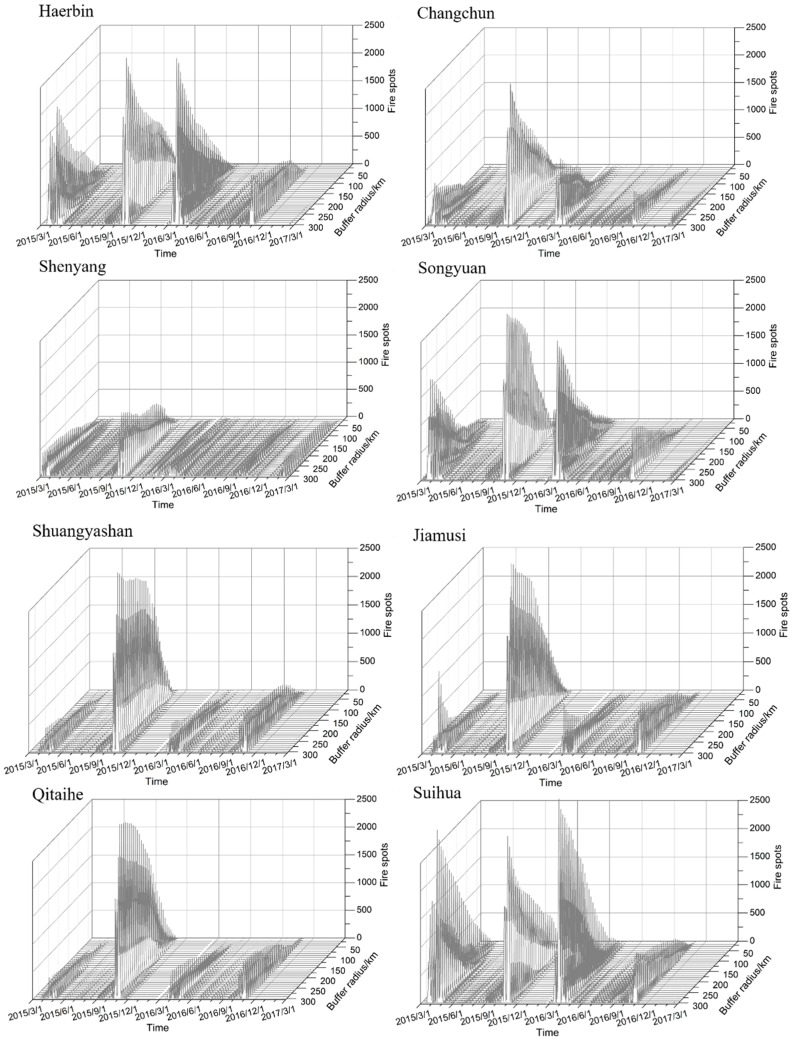
Variations in the number of fire spots in different buffers of a typical city (the x-axis is the time axis, the y-axis is the buffer radius, and the z-axis is the number of fire spots).

The change in the number of fire spots in different buffers of a typical city (Figure 10) shows that the number of fire spots in spring and autumn is the highest. For the three provincial capital cities, the number of fire spots is in the following order: Harbin > Changchun > Shenyang; considering the typical urban distribution latitude, the number of fires in the north of a city is significantly higher than that in the south. Additionally, for the typical cities in the northeastern part of Heilongjiang, such as Shuangyashan, Jiamusi and Qitaihe, the number of fire points in autumn is much higher than that in spring. The distribution of fire points over time in different radii of these cities is highly consistent. In a straw burning year, except for in Suihua, the number of typical city straw-burning points reached its highest value in the autumn of 2015.

#### 3.5.2. Changes in the Typical Urban Pollution Index

**Figure 11:**
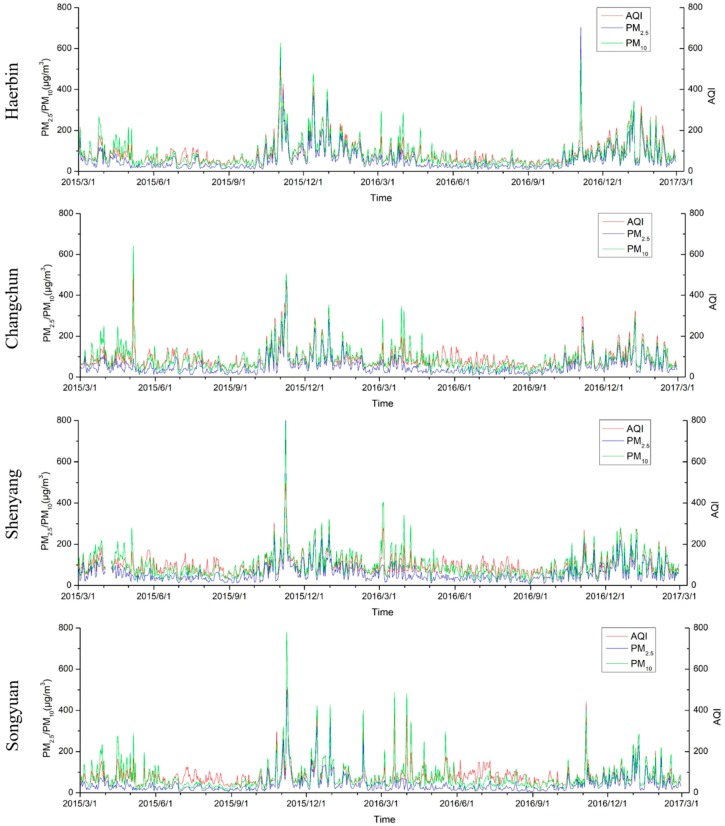

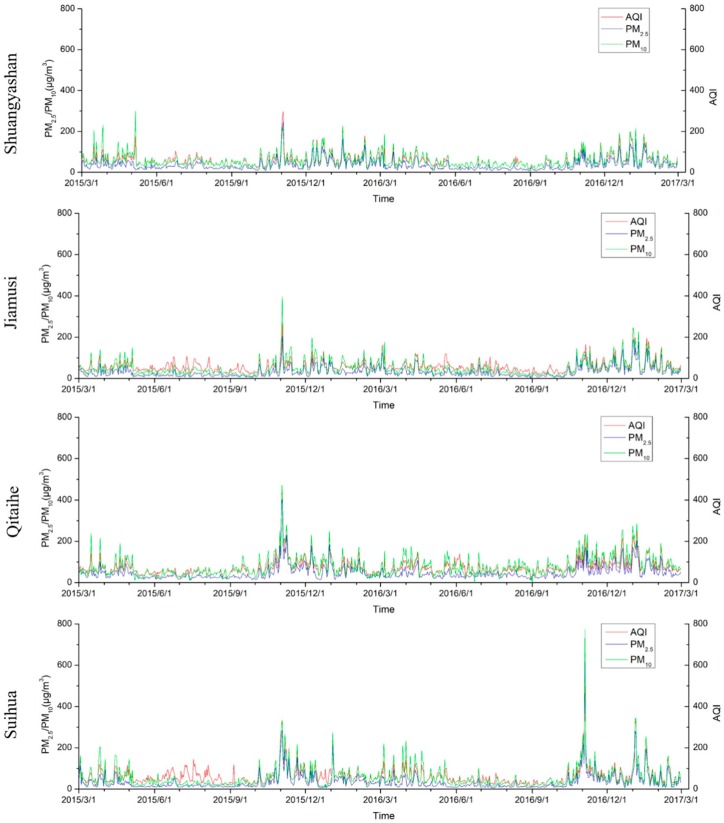
Changes in the typical urban pollution index from 2015 to 2016.

The 2015–2016 typical urban pollution index change chart (Figure 11) shows that, in 2015, the highest peak of the pollution index in each city occurred in early November, followed by peak times in early and late December. In addition to peaks in the first half of January of the following year, there were also smaller peaks in the March–May period of 2015; in 2016, except for the peak times in the Harbin, Songyuan and Suihua pollution indexes, which occurred in early November, the peaks in other cities were not obvious. The pollutant concentration was high from December to January, and there were many small peaks from March–April 2016. The peak value in March 2016 was slightly higher than of the peak in March 2015. Notably, the pollutant concentrations in Changchun, Songyuan, Shenyang and Harbin in March and April 2016 were more serious than those in 2015; the difference from December 2015 was not large.

#### 3.5.3. Relationship between the Number of Fire Spots and The Pollutant Concentration in Different Buffer Zones of Typical Cities

**Figure 12:**
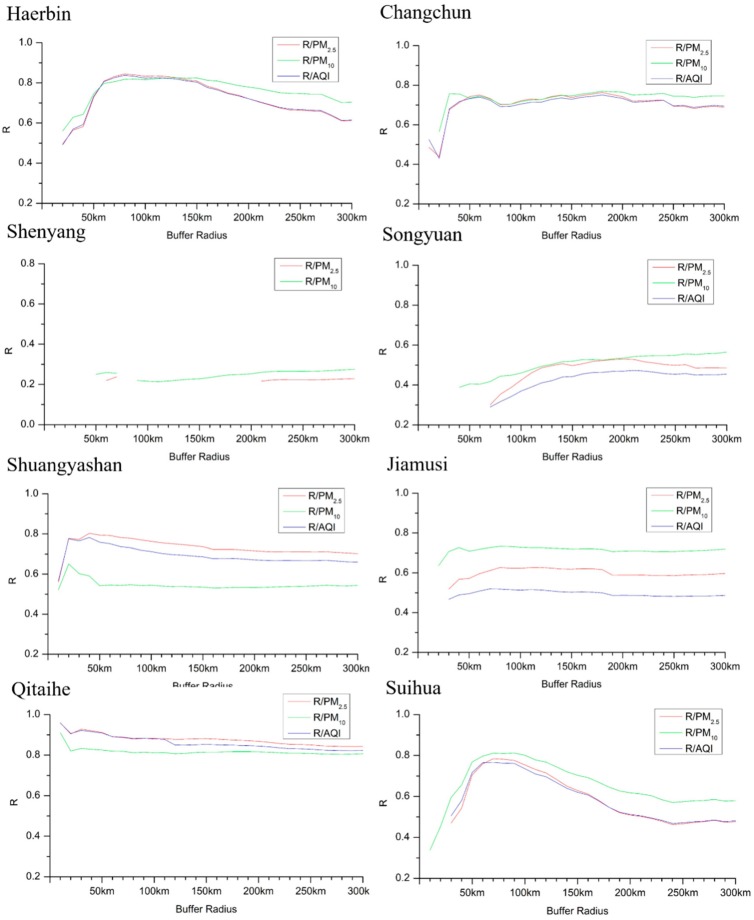
Relationship between the number of fire spots and the pollutant concentration in different buffer zones of typical cities.

Considering the combination of temporal and spatial factors, there are also peaks in the spring and autumn corresponding to the number of typical city straw fire spots. The pollutants PM_2.5_, PM_10_, and AQI and the number of fire spots in different radii of the typical cities, except for Shenyang, have a maximum correlation coefficient *R* of 0.7 or greater (*p* < 0.05), as shown in Figure 12. Figure 12 shows the relationship between the number of fire spots and the pollutant concentration *R* in the different buffer radii of a typical city as a function of the radius at *p* < 0.05.

Among these cities, from the change of the correlation coefficient *R* value with the radius, both Harbin and Suihua have a trend of increasing first and then decreasing, which has strong consistency. Changchun decreases first at 0–20 km and increases at 20 km–50 km. After 50 km, the correlation coefficient *R* value is basically unchanged at approximately 0.7. The correlation coefficient *R* of Shenyang shows a tendency to increase slowly with the radius. The *R* values of Songyuan and Jiamusi tend to be stable after a slow increase with the radius. Shuangyashan appears to increase first and then decrease, and finally, the *R* value remains basically unchanged. The correlation coefficient of Qitaihe has the largest *R* value, and the overall trend tends to remain unchanged after it first decreases. The correlation coefficient *R* values of Changchun, Songyuan, Jiamusi, Shuangyashan and Qitaihe all tend to remain basically unchanged as the radius becomes larger. Since some correlation coefficient *R* values do not satisfy the condition of *p* < 0.05, there is data missing in the fold line in Figure 12.

#### 3.5.4. Analysis of the Impact of Straw Burning on Urban Pollution in Typical Cities

By analysing the correlation between the number of fire spots with different radii in typical cities and urban PM_2.5_, under the premise of *p* < 0.05, we fitted and analysed the curve of the correlation coefficient *R* with the radius to obtain the functional relationship between them. The function formula is shown in Table 3. The curve of the correlation coefficient *R* and the radius of Songyuan is well fitted, and there is an obvious positively correlated relationship. The fitting curve of the *R* values and radii of Harbin and Suihua shows that *R* is positively correlated with a radius in the range of 80 km and negatively correlated with those more than 80 km (Figure 12). This finding indicates that the effect of straw burning on PM_2.5_ has a positive effect in the range of 80 km, while in the range of more than 80 km, the effect of straw burning on PM_2.5_ is gradually weakened. The curve fitting of the *R* values and the radii of Changchun and Jiamusi shows that the *R* values are positively correlated with the radius in the range of 20 to 70 km and then are basically in a stable state, indicating that the level of PM_2.5_ in the range of 20 to 70 km is greatly affected by straw burning. The *R* value of Songyuan increased with the increase of the radius at 70–200 km, indicating that PM_2.5_ is greatly affected by straw burning in this range. The *R* values and radii of Shuangyashan and Qitaihe show a negative correlation with the radius, indicating that in the range of more than 20 km, the smaller the radius, the greater the impact of straw burning on PM_2.5_.

We selected the radius corresponding to the maximum R value obtained by the correlation analysis between the typical city PM_2.5_ and the number of fire spots in different radii and determined the fitting function, as shown in Table 4. The number of fire spots in the seven typical cities is linear with the buffer radius and is positively correlated. Among these cities, the maximum R value was found in Qitaihe, and the lowest was observed in Songyuan. The buffer radius corresponding to the maximum R value radius of Qitaihe, Shuangyashan, Harbin and Suihua is within 100 km. The buffer radius corresponding to the maximum R value radius of Changchun, Jiamusi and Songyuan is within 100–200 km. The results of typical urban studies show that the radius of influence of straw burning on urban pollutant concentration differs in different cities. Varied policies can be adopted to control straw burning and pollutants according to the different characteristics of each city. The straw burning zone and the limited burning zone have great reference value and practical significance.

## 4. Discussion

### 4.1. Influence of Straw Burning on Urban Air Pollutant Concentrations

The correlation coefficient between the number of fire spots in the 10–300 km radius buffer zone and the urban centre PM_2.5_, PM_10_ and AQI levels in 41 cities in Northeast China was calculated. We found that the results of the cluster analysis of the radii of PM_2.5_ and PM_10_ are completely consistent. Among them, the radii of 10 km, 20 km and 30 km are each of the same type; the radius of 40 km to 60 km is one type, the radius of 70 km–150 km is one type, and the radius of 160 km–300 km is one type. AQI clusters of radii are divided into five categories. Of these categories, the radii of 10 km and 20 km are each one class, the radius of 30 km to 60 km is one class, the radius of 70 km–200 km is one class, and the radius of 210 km–300 km is one class. The effect of straw burning on pollutants has a certain relationship with the radius, but there is no uniform radius threshold in different cities. This finding is inconsistent with the results of the good correlation between the change trend of fire spots in the buffer zone of 700 to 800 km in a city in previous studies and the air pollution index [14]. In the city cluster analysis, cities with large correlation coefficient *R* values are aggregated. Cities with smaller *R* values are distributed on the periphery of cities with the largest *R* values. The cities with the smallest *R* values are distributed at the outermost periphery. These cities are in a clustered distribution state with the *R* value.

### 4.2. Relationship between the Number of Fires and Pollutants in Rice and Corn Concentrated Planting Areas

In cities with a concentration coefficient of rice and corn greater than 1 in the northeastern region, the number of straw burning fire spots in the same buffer zone is higher than that in other cities. In cities with a rice or corn concentration coefficient greater than 1 in Northeast China, the correlation coefficient between the number of fire points and PM_2.5_, PM_10_ and AQI in most cities is greater than 0.5 (*p* < 0.05). It is indicated that the more concentrated rice and corn are planted around the city in Northeast China, the greater the amount of straw, the more fire spots generated by rice or corn stover burning and the greater the impact of the generated pollutants on the urban pollutant concentration.

### 4.3. Relationship between the Number of Fire Spots and Pollutants in Typical Cities

The quantitative analysis of the eight selected typical cities shows that the impact radius of straw burning varies in different cities. The radius of the buffer corresponding to the maximum *R* values in Qitaihe, Shuangyashan, Harbin and Suihua is within 100 km. The radius of the buffer corresponding to the maximum *R* values in Changchun, Jiamusi and Songyuan is within 100–200 km. The impact radius of straw burning on urban pollutants varies in different cities. Varied policies can be adopted to control straw burning and pollutants according to different city characteristics.

### 4.4. Other Factors Influencing Urban Air Pollutant Concentrations

One of the main causes of urban pollutant concentrations is the pollution source. In addition to burning straw in Northeast China, there are some effects from automobile exhaust, winter heating (October–April of the following year) and factories. The effects of other sources of pollution were not considered in this study. The capital cities in Northeast China are densely populated, and there are many heavy industries that produce a large amount of industrial exhaust and automobile exhaust.

Meteorological conditions are also an important factor in changing pollutant concentrations in urban cities. In windy weather, pollutants are quickly blown away by the wind, reducing pollutant concentrations. Wang et al. studied the effects of wheat straw burning on regional aerosols in northern China. The results show that the average AOD and PM_10_ concentrations in the North Plain vary greatly with wind direction and humidity [11].

## 5. Conclusions

The fire spots of straw burning in Northeast China are concentrated in spring and autumn. The fire spots in spring are mainly distributed in southwestern Heilongjiang Province. The fire spots in autumn are mainly distributed in northeastern Heilongjiang Province, northwestern and central Jilin Province, and central Liaoning Province. From 2013–2016, for the fire spots of straw burning in northeastern China in the northeastern region, the level of PM_2.5_, PM_10_ and AQI in 2015–2016 was higher than that in winter and peaked in spring and autumn. We speculate that straw burning promotes pollutant generation to some extent. We used 41 cities in Northeast China as the centres and made buffer zones with radii increasing every 10 km. The correlation coefficient *R* values of PM_2.5_, PM_10_ and AQI in the central cities and the number of fire spots in different buffers were calculated, respectively. According to the variation of the *R* values with the radii, 41 cities were clustered and analysed. We found that the cities were clustered and distributed in regions with different *R* values. Radius cluster analysis shows that adjacent radii are clustered into one class. In cities with a concentration factor of rice and corn planting greater than 1 in Northeast China, the correlation coefficient of the number of fire spots and pollutants is generally higher.

The main innovations and contributions of this research are: A quantitative study on the temporal and spatial distribution characteristics of fire spots and pollutants in Northeast China that was carried out to provide a reference for studying the relationship between straw and pollutants. By clustering the relationship between pollutants and straw burning, the geospatial law of the effects of straw burning on pollutants has been revealed.

## Figures and Tables

**Table 1:** MOD14A1 and MYD14A1 FireMask data value description.

Value	Description
0	Not processed (missing input data)
1	Not processed (obsolete; not used since Collection 1)
2	Not processed (other reason)
3	Non-fire water pixel
4	Cloud (land or water)
5	Non-fire land pixel
6	Unknown (land or water)
7	Fire (low confidence, land or water)
8	Fire (nominal confidence, land or water)
9	Fire (high confidence, land or water)

**Table 2:** Codes for 41 cities or regions in Northeast China.

City	Code	City	Code	City	Code
Heihe	1	Baicheng	15	Fuxin	29
Daxinganlingdiqu	2	Songyuan #	16	Chifeng	30
Hulunbeier	3	Mudanjiang	17	Chaoyang	31
YIchun	4	Yanji	18	Benxi	32
Hegang	5	Jilin	19	Liaoyang	33
Jiamusi #	6	Changchun #	20	Anshan	34
Shuangyashan #	7	Tongliao	21	Panjin	35
Qitaihe #	8	Siping	22	Jinzhou	36
Jixi	9	Liaoyuan	23	Huludao	37
Suihua #	10	Baishan	24	Yingkou	38
Harbin #	11	Tonghua	25	Dandong	39
Daqing	12	Tieling	26	Wafangdian	40
Qiqihaer	13	Fushun	27	Dalian	41
Wulanhaote	14	Shenyang #	28		

# is a typical city.

**Table 3:** Relationship between the number of fire spots in different buffers of a typical city and the correlation coefficient *R* of urban PM_2.5_ with the radii.

City	Formula	*R*-Square
Haerbin	y = 10^−4^x^3^ − 0.0066x^2^ + 0.1064x + 0.3202	0.9249
Changchun	y = 7 × 10^−7^x^5^ − 6 × 10^−5^x^4^ + 0.0019x^3^ − 0.0271x^2^ + 0.1811x + 0.287	0.8284
Shengyang	y = 6 × 10^−5^x^3^ − 0.0011x^2^ + 0.0063x + 0.2108	0.9706
Songyuan	y = −0.001x^2^ + 0.0409x + 0.1037	0.8987
Shuangyashan	y = 5 × 10^−7^x^5^ − 5 × 10^−5^x^4^ + 0.0015x^3^ − 0.0229x^2^ + 0.1435x + 0.4955	0.7987
Jiamusi	y = −3 × 10^−6^x^4^ + 0.0002x^3^ − 0.0067x^2^ + 0.0778x + 0.3247	0.944
Qitaihe	y = −10^−5^x^3^ + 0.0006x^2^ − 0.0112x + 0.9494	0.9066
Suihua	y = 2 × 10^−4^x^3^ − 0.0084x^2^ + 0.1158x + 0.265	0.9027

**Table 4:** The number of fire points in the radius with the largest *R* value and the PM_2.5_ fitting function.

City	Maximum *R* Value/PM_2.5_	Corresponding Buffer Radius/km	Formula
Haerbin	0.844	80	y = 0.6299x + 227.92
Changchun	0.763	180	y = 0.3407x + 311.98
Shengyang	0.238	70	y = 0.3719x + 426.05
Songyuan	0.53	200	y = 0.0845x + 220.20
Shuangyashan	0.803	40	y = 0.5837x + 225.12
Jiamusi	0.627	120	y = 0.1169x + 172.79
Qitaihe	0.959	10	y = 40.266x + 179.97
Suihua	0.784	70	y = 0.8985x + 131.24
